# Urinary Bisphenol A Levels and Measures of Obesity: Results from the National Health and Nutrition Examination Survey 2003–2008

**DOI:** 10.5402/2012/965243

**Published:** 2012-07-18

**Authors:** Anoop Shankar, Srinivas Teppala, Charumathi Sabanayagam

**Affiliations:** ^1^Department of Community Medicine, West Virginia University School of Medicine, Robert C. Byrd Health Sciences Center, 1 Medical Center Drive, P.O. Box 9190, Morgantown, WV 26505-9190, USA; ^2^Singapore Eye Research Institute, Singapore 168751; ^3^Department of Clinical Sciences, Duke-NUS Graduate Medical School, Singapore 169857

## Abstract

Bisphenol A (BPA) is a widely used chemical. We examined the association between urinary BPA levels and obesity in the National Health and Nutritional Examination Survey (NHANES) 2003–2008. The main outcome of interest was obesity defined as (1) body mass index (BMI) ≥ 30 Kg/m^2^ and (2) waist circumference (WC) ≥ 102 cm in men and ≥ 88 cm in women. Urinary BPA levels were examined in quartiles. Overall, we observed a positive association between increasing levels of urinary BPA and both measures of obesity, independent of potential confounding factors including, smoking, alcohol consumption, and serum cholesterol levels. Compared to quartile 1 (referent), the multivariate-adjusted odds ratio (95% confidence interval) associated with quartile 4 for BMI-based obesity was 1.69 (1.30–2.20); *P*-trend < 0.0001 and for WC-based obesity was 1.59 (1.21–2.09); *P*-trend = 0.0009. This association between BPA and both measures of obesity was consistently present across gender and race-ethnic groups (all *P*-trend < 0.05). Elevated levels of urinary BPA are associated with measures of obesity independent of traditional risk factors. This association is consistently present across gender and race-ethnic groups. Future prospective studies are needed to confirm or disprove this finding.

## 1. Introduction

As obesity is a major public health problem in the US [1] and a strong risk factor for cardiovascular disease and cancer, it is important to identify common environmental risk factors that may have a role in weight gain and the development of obesity. Bisphenol A (BPA) is a chemical produced in high volumes worldwide and used extensively in the manufacture of epoxy resins, polycarbonate plastics, and food and beverage containers [2]. With this one of the commonly exposed environmental chemicals in humans, recent biomonitoring surveys have demonstrated that detectable levels of BPA in urine are present in nearly all sampled adults in the US [3]. Recent evidence mostly from animal studies has suggested that BPA exposure may be related to increased insulin resistance [4] and therefore may have a role in weight gain and the development of obesity [5, 6]. Also, BPA exposure has been shown to be associated with high lipid levels [7] and increased levels of serum markers of oxidative stress [8] and inflammation [9] all of which are mechanisms that have been implicated in the development of obesity. Therefore, it is intriguing to hypothesize that environmental exposure to BPA may have a role in the development of obesity.

To date, only one study in humans has examined the putative association between BPA exposure and obesity, and it reported an overall positive association between urinary BPA levels and obesity in the National Health and Nutrition Examination Survey [10]. However, several questions related to this hypothesis remain unanswered. First, BPA is known to be an estrogen agonist [4], and it is not known whether the effect of BPA on obesity is similarly present in the men as well as women. Second, previous reports have shown that there are racial/ethnic variations in BPA exposure in the US general population samples [3], and therefore it needs to be studied whether the effect of BPA on obesity is consistently present in various racial/ethnic groups. In this context, we combined six years of contemporary NHANES data, which gave us enough sample size and statistical power, to examine the association between urinary BPA levels and obesity by gender and race/ethnicity in a population-based sample of US adults.

## 2. Methods

The current study is based on data from the NHANES 2003–2008. Detailed description of NHANES study design and methods is available elsewhere [11–13]. In brief, the NHANES survey included a stratified multistage probability sample representative of the civilian noninstitutionalized US population. Selection was based on counties, blocks, households, and individuals within households and included the oversampling of non-Hispanic blacks and Mexican Americans in order to provide stable estimates of these groups. Subjects were required to sign a consent form before their participation, and approval was obtained from the Human Subjects Committee in the US Department of Health and Human Service.

The current study sample consisted of participants aged greater than 20 years among whom urinary BPA was available (*n = *4,792). We further excluded subjects with self-reported cardiovascular disease (*n = *495) and also subjects with missing data (*n = *330) on covariates included in the multivariable model, including level of education, smoking status, serum glucose levels, systolic or diastolic blood pressure, body mass index (BMI), or cholesterol levels including 3,967 participants (51.7% women) in the final analysis. 

### 2.1. Exposure Measurements

Age, gender, race/ethnicity, smoking status, alcohol intake (g/day), level of education, history of diabetes and oral hypoglycemic intake or insulin administration were assessed using a questionnaire [11–13]. Individuals who had not smoked >100 cigarettes in their lifetimes were considered never smokers; those who had smoked >100 cigarettes in their lifetimes were considered former smokers if they answered negatively to the question “Do you smoke now?” and current smokers if they answered affirmatively. Moderate physical activity was defined as engaging in moderate-intensity sports, fitness, or recreational activities that cause a small increase in breathing or heart rate such as brisk walking, bicycling, swimming, or golf for at least 10 minutes continuously in a typical week. Rigorous procedures with quality control checks were used in blood collection, and details about these procedures are provided in the NHANES Laboratory/Medical Technologists Procedures Manual [11–13]. Seated systolic and diastolic blood pressures were measured using a mercury sphygmomanometer according to the American Heart Association and Seventh Joint National Committee (JNC7) recommendations [14]. Up to 3 measurements were averaged for systolic and diastolic pressures. Patients were considered hypertensive if they reported current blood pressure-reducing medication use and/or had systolic blood pressures >140 mm of Hg and/or diastolic blood pressures >90 mm of Hg. Diabetes mellitus was defined based on the recent guidelines of the American Diabetes Association [15] as a serum glucose >126 mg/dL after fasting for a minimum of 8 hours, a serum glucose >200 mg/dL for those who fasted <8 hours before their NHANES visit, a glycosylated hemoglobin value >6.5%, or a self-reported current use of oral hypoglycemic medication or insulin.

Previous measures of BPA in biological matrixes involved techniques such as gas chromatography (GC) or high-performance liquid chromatography [16]. To achieve enhanced sensitivity and selectivity over previous methods, in the current NHANES, measures of environmental phenols were derivatized to alkyl or acyl derivatives before GC-mass spectrometry (GC/MS) analysis [17]. Using solid phase extraction coupled to high-performance liquid chromatography and tandem mass spectrometry, detection levels of 0.1–2 nanograms per milliliter (ng/mL) in 100 *μ*L of urine were achieved, sufficient for measuring urinary BPA levels in nonoccupationally exposed participants [17]. 

### 2.2. Main Outcome of Interest: Obesity

Height, weight, and waist circumference were measured by trained technicians during the MEC examination using standardized protocol [18]. Height was measured without shoes to the nearest 0.1 cm using a stadiometer. Weight was measured in kilograms using a digital scale. BMI was calculated as weight in kilograms divided by height in meters squared. WC was measured to the nearest 0.1 cm. Obesity was defined as (1) a body mass index (BMI) of ≥30 kg/m^2^ and (2) waist circumference (WC) ≥ 102 cm in men and ≥ 88 cm in women [19].

### 2.3. Statistical Analysis

Urinary BPA was categorized into quartiles (<1.10 ng/mL, 1.10–2.10 ng/mL, 2.11–4.20 ng/mL, and >4.20 ng/mL). We hypothesized that high BPA levels are associated with measures of obesity. The odds ratio ((OR) (95% confidence interval (CI))) of obesity for BPA was calculated by taking the lowest quartile (quartile 1) as the referent, using multivariable logistic regression models. We used two models: the age- and sex-adjusted model, the multivariable model, additionally adjusting for race/ethnicity (non-Hispanic whites, non-Hispanic blacks, Mexican Americans, and others), education categories (below high school, high school, above high school), smoking (never smoker, former smoker, current smoker), alcohol intake (nondrinker, moderate drinker, heavy drinker), physical inactivity (absent, present), diabetes (absent, present), hypertension (absent, present), and total serum cholesterol (mg/dL). Trends in the OR of obesity measures across increasing urinary BPA categories were determined by modeling BPA as an ordinal variable. Sample weights that account for the unequal probabilities of selection, oversampling, and nonresponse were applied for all analyses using SAS (version 9.2; SAS Institute, Cary, NC) and SUDAAN software; SEs were estimated using the Taylor series linearization method.

## 3. Results

Table 1 presents the baseline characteristics of the study population by gender. 52.6% of the study population were women. 

Table 2 presents the association between urinary BPA levels and obesity, defined by BMI, in the whole population, as well as by gender and race/ethnicity. We observed a positive one between BPA levels and obesity in both the age, sex-adjusted model as well as the multivariable-adjusted model in the whole population as well as in men, women, non-Hispanic whites, non-Hispanic blacks, Mexican Americans, and others.

Similar to the previous table, Table 3 presents the association between urinary BPA levels and obesity, defined by waist circumference, in the whole population as well as by gender and race ethnicity. Here also, we observed a consistent positive association between BPA levels and obesity in the whole population as well as in men, women, non-Hispanic whites, non-Hispanic blacks, Mexican Americans, and others.

## 4. Discussion

In a contemporary general population sample of US adults, we found that higher urinary BPA positively associated with obesity in the whole population as well as in subgroup analysis by gender and race/ethnicity. The association appeared to be independent of confounding factors including age, education, smoking, alcohol intake, physical activity, and other factors. The association between BPA and obesity was consistently present when we defined obesity by BMI as well as by waist circumference. Our study adds to the existing literature on the association between BPA exposure and obesity by suggesting that the previously observed association was consistently present in both genders as well as in all major racial/ethnic groups in the US general population.

An association between exposure to BPA and obesity is biologically plausible. Alonso-Magdalena et al. reported that mice treated with low-dose BPA developed hyperinsulinemia and insulin resistance [4]. Hugo et al. have suggested that BPA exposure is associated with lower adiponectin levels and elevated levels of inflammatory cytokines [20]. In experimental animal studies, in utero exposure to BPA was found to be associated with greater weight in adolescence and early adulthood [5].

Previous studies on the association between BPA levels and obesity in humans have not been entirely consistent. Lang et al. did not find an association between BPA levels and obesity [21] whereas Carwile and Michels reported a positive association between BPA and obesity in the US [10]. Takeuchi et al. reported positive association in a small sample of Japanese women [22], and Wang et al. reported a positive association in Chinese adults [23]. Our study adds to the existing literature on the positive association between BPA exposure and obesity. Also, similar to Carwile et al. [10] we have showed that the association between BPA and obesity was consistently present when we used BMI as well as waist circumference as the measure of adiposity.

Since BPA levels have been reported to vary by gender and race/ethnicity, we examined BPA-obesity association within subgroups of these factors. We found that the BPA-obesity association was consistently present in men as well as women, suggesting that even though BPA is known to be an estrogen agonist, its effect on weight gain and obesity within each gender is probably independent of female hormones. Similarly within subgroups of race/ethnicity, we found that there was a consistent positive association between BPA and obesity within these subgroups also.

The advantages of our study include its population-based nature, large sample size, and the availability of data on several confounders. The main study limitation is the cross-sectional nature of the current study which precludes any conclusions on our part regarding the causal nature of the observed association between BPA levels and obesity. In this context, reverse causality is a possibility as BPA is known to be lipophilic and also has been shown to be stored in adipose tissues [24]. However, an argument against the reverse causality hypothesis is that Fernandez et al. did not find any positive association between BMI and adipose tissue BPA concentration [25]. We believe that more longitudinal, studies examining the putative association between BPA levels and obesity are needed before any conclusions can be made. 

In summary, in a population-based sample of US adults, we found that higher urinary BPA levels are positively associated with obesity in the whole population as well as in subgroup analysis by gender and race/ethnicity. The association between BPA and obesity was consistently present when we defined obesity by BMI as well as by waist circumference.

## Figures and Tables

**Table 1:** Baseline characteristics of the study population, by gender^∗^.

Characteristics	Men (*n* = 1879)	Women (*n* = 2088)
Age (years)	44.3 ± 0.5	45.6 ± 0.4
Race/ethnicity (%)		
Non-Hispanic Whites	953 (71.5)	991 (70.0)
Non-Hispanic Blacks	373 (9.7)	425 (11.5)
Mexican Americans	351 (8.5)	436 (8.0)
Others	202 (10.2)	236 (10.4)
Education categories (%)		
Below high school	511 (17.9)	560 (16.7)
High school	476 (25.5)	510 (24.9)
Above high school	892 (56.6)	1018 (58.4)
Smoking (%)		
Never smoker	840 (45.7)	1289 (58.7)
Former smoker	529 (27.2)	403 (20.3)
Current smoker	510 (27.1)	396 (21.0)
Alcohol intake (%)		
Nondrinker	533 (24.5)	894 (35.7)
Moderate drinker	663 (38.7)	863 (47.3)
Heavy drinker	683 (36.8)	331 (17.0)
Physical inactivity %	1246 (60.3)	1365 (59.5)
Body mass index (BMI) (%)		
Nonobese (<30 kg/m^2^)	1270 (66.7)	1313 (66.7)
Obese (BMI ≥ 30 kg/m^2^)	609 (33.3)	775 (33.2)
Systolic blood pressure (mm of Hg)	123.1 ± 0.4	119.1 ± 0.5
Diastolic blood pressure (mm of Hg)	72.4 ± 0.4	69.2 ± 0.3
Total cholesterol (mg/dL)	201.55 ± 1.12	202.23 ± 1.03
Urinary bisphenol A (ng/mL)	3.97 ± 0.21	3.90 ± 0.26
Diabetes (%)	229 (9.5)	238 (8.7)

^∗^Data presented are numbers (percentages) or mean values ± standard error (SE), as appropriate for the variable.

**Table 2:** Association between urinary bisphenol A and obesity (body mass index ≥ 30 kg/m^2^).

Bisphenol A quartiles (ng/mL)	Sample size (obesity %)	Age, sex-adjusted OR (95% CI)^∗^	Multivariable-adjusted OR (95% CI)^∗†^
Whole Population			
Quartile 1 (<1.10)	1121 (26.2)	1 (referent)	1 (referent)
Quartile 2 (1.10–2.10)	905 (33.6)	1.46 (1.18–1.80)	1.40 (1.10–1.76)
Quartile 3 (2.11–4.20)	977 (36.3)	1.67 (1.31–2.12)	1.59 (1.25–2.02)
Quartile 4 (>4.20)	964 (38.1)	1.81 (1.41–2.33)	1.69 (1.30–2.20)
*P*-trend		<0.0001	<0.0001
Men			
Quartile 1 (<1.10)	460 (27.8)	1 (referent)	1 (referent)
Quartile 2 (1.10–2.10)	455 (33.4)	1.33 (1.00–1.77)	1.32 (0.95–1.82)
Quartile 3 (2.11–4.20)	489 (36.8)	1.59 (1.08–2.34)	1.56 (1.04–2.34)
Quartile 4 (>4.20)	475 (34.8)	1.47 (1.06–2.03)	1.45 (1.04–2.02)
*P*-trend		0.02	0.03
Women			
Quartile 1 (<1.10)	661 (25.1)	1 (referent)	1 (referent)
Quartile 2 (1.10–2.10)	450 (33.8)	1.54 (1.10–2.15)	1.43 (1.01–2.03)
Quartile 3 (2.11–4.20)	488 (35.8)	1.67 (1.26–2.26)	1.59 (1.16–2.18)
Quartile 4 (>4.20)	489 (41.7)	2.17 (1.56–3.03)	1.93 (1.35–2.74)
*P*-trend		<0.0001	0.0001
Non-Hispanic whites			
Quartile 1 (<1.10)	603 (25.7)	1 (referent)	1 (referent)
Quartile 2 (1.10–2.10)	443 (33.5)	1.45 (1.09–1.94)	1.38 (0.99–1.89)
Quartile 3 (2.11–4.20)	473 (35.1)	1.56 (1.14–2.19)	1.46 (1.04–2.05)
Quartile 4 (>4.20)	425 (36.3)	1.68 (1.20–2.36)	1.52 (1.05–2.18)
*P*-trend		0.002	0.02
Non-Hispanic blacks			
Quartile 1 (<1.10)	149 (39.9)	1 (referent)	1 (referent)
Quartile 2 (1.10–2.10)	152 (43.4)	1.31 (0.77–2.22)	1.35 (0.78–2.32)
Quartile 3 (2.11–4.20)	227 (49.3)	1.59 (1.02–2.48)	1.78 (1.07–2.97)
Quartile 4 (>4.20)	270 (47.7)	1.48 (1.00–2.23)	1.60 (1.02–2.51)
*P*-trend		0.05	0.03
Mexican Americans and others			
Quartile 1 (<1.10)	369 (23.4)	1 (referent)	1 (referent)
Quartile 2 (1.10–2.10)	310 (30.3)	1.48 (0.98–2.22)	1.44 (0.94–2.19)
Quartile 3 (2.11–4.20)	277 (32.1)	1.64 (1.12–2.39)	1.59 (1.12–2.27)
Quartile 4 (>4.20)	269 (36.2)	2.01 (1.26–3.20)	1.97 (1.24–3.12)
*P*-trend		0.002	0.002

^∗^OR (95% CI): Odds Ratio (95% Confidence Interval)

^†^Adjusted for age (years), gender (male, female), race ethnicity (non-Hispanic whites, non-Hispanic blacks, Mexican Americans, and others), education categories (below high school, high school, above high school), smoking (never, former, current), alcohol intake (nondrinker, moderate drinker, heavy drinker), physical inactivity (absent, present), diabetes (absent, present), hypertension (absent, present), and total cholesterol (mg/dL).

**Table 3:** Association between urinary bisphenol A and obesity (waist circumference: men ≥ 102 cm; women ≥ 88 cm).

Bisphenol A quartiles (ng/mL)	Sample size (obesity %)	Age, sex-adjusted OR (95% CI)^∗^	Multivariable-adjusted OR (95% CI)^∗†^
Whole Population			
Quartile 1 (<1.10)	1101 (46.1)	1 (referent)	1 (referent)
Quartile 2 (1.10–2.10)	885 (54.2)	1.66 (1.25–2.19)	1.63 (1.20–2.22)
Quartile 3 (2.11–4.20)	958 (53.1)	1.71 (1.33–2.21)	1.66 (1.28–2.14)
Quartile 4 (>4.20)	938 (52.2)	1.72 (1.33–2.22)	1.59 (1.21–2.09)
*P*-trend		<0.0001	0.0009
Men			
Quartile 1 (<1.10)	451 (36.8)	1 (referent)	1 (referent)
Quartile 2 (1.10–2.10)	442 (46.1)	1.58 (1.18–2.10)	1.62 (1.18–2.23)
Quartile 3 (2.11–4.20)	478 (43.6)	1.56 (1.07–2.27)	1.55 (1.05–2.29)
Quartile 4 (>4.20)	457 (42.3)	1.54 (1.11–2.15)	1.56 (1.08–2.24)
*P*-trend		0.03	0.05
Women			
Quartile 1 (<1.10)	650 (52.6)	1 (referent)	1 (referent)
Quartile 2 (1.10–2.10)	443 (62.2)	1.67 (1.12–2.49)	1.60 (1.03–2.48)
Quartile 3 (2.11–4.20)	480 (63.1)	1.81 (1.31–2.50)	1.74 (1.24–2.44)
Quartile 4 (>4.20)	481 (62.3)	1.84 (1.27–2.69)	1.64 (1.11–2.42)
*P*-trend		0.0005	0.005
Non-Hispanic whites			
Quartile 1 (<1.10)	593 (47.4)	1 (referent)	1 (referent)
Quartile 2 (1.10–2.10)	431 (57.3)	1.73 (1.23–2.42)	1.69 (1.16–2.47)
Quartile 3 (2.11–4.20)	466 (52.9)	1.57 (1.13–2.19)	1.49 (1.06–2.09)
Quartile 4 (>4.20)	409 (52.2)	1.61 (1.17–2.23)	1.46 (1.03–2.06)
*P*-trend		0.007	0.04
Non-Hispanic blacks			
Quartile 1 (<1.10)	147 (53.9)	1 (referent)	1 (referent)
Quartile 2 (1.10–2.10)	148 (48.9)	1.17 (0.74–1.84)	1.21 (0.73–1.98)
Quartile 3 (2.11–4.20)	221 (64.4)	2.10 (1.27–3.48)	2.39 (1.39–4.12)
Quartile 4 (>4.20)	265 (55.0)	1.34 (0.88–2.07)	1.43 (0.92–2.22)
*P*-trend		0.12	0.07
Mexican Americans and others			
Quartile 1 (<1.10)	361 (38.4)	1 (referent)	1 (referent)
Quartile 2 (1.10–2.10)	306 (46.1)	1.64 (1.06–2.53)	1.55 (0.98–2.46)
Quartile 3 (2.11–4.20)	271 (45.7)	1.76 (1.03–2.99)	1.72 (1.00–2.95)
Quartile 4 (>4.20)	264 (49.4)	2.19 (1.36–3.53)	2.05 (1.25–3.35)
*P*-trend		0.001	0.003

^∗^OR (95% CI): Odds Ratio (95% Confidence Interval)

^†^Adjusted for age (years), gender (male, female), race ethnicity (non-Hispanic whites, non-Hispanic blacks, Mexican Americans, and others), education categories (below high school, high school, above high school), smoking (never, former, current), alcohol intake (nondrinker, moderate drinker, heavy drinker), physical inactivity (absent, present), diabetes (absent, present), hypertension (absent, present), and total cholesterol (mg/dL).

## List of Abbreviations

BMI: Body mass index
WC: Waist circumference
BPA: Bisphenol-A
CVD: Cardiovascular disease
NHANES: National Health and Nutrition Examination Survey.
